# The future of fertility healthcare in South Africa

**DOI:** 10.3389/frph.2026.1770259

**Published:** 2026-03-13

**Authors:** Donrich W. Thaldar, Nomfundo N. Mthembu

**Affiliations:** School of Law, University of KwaZulu-Natal, Durban, South Africa

**Keywords:** donor practices, fertility tourism, *in vitro* fertilisation, patient-centred care, regulatory oversight

## Abstract

**Introduction:**

The South African fertility healthcare sector offers world-class clinical expertise, advanced infrastructure, and increasing international appeal. Despite these strengths, the sector faces persistent challenges related to access, regulatory enforcement, and workforce capacity. This study aimed to analyse the sector's strategic position and identify key areas for development.

**Methods:**

Semi-structured interviews were conducted with twelve leaders in the fertility healthcare sector. Data were analysed using a SWOT (Strengths, Weaknesses, Opportunities, Threats) framework to evaluate internal and external factors influencing the sector.

**Results:**

Stakeholders identified key strengths, including the availability of donors from diverse racial and ethnic backgrounds and the relative affordability of high-quality treatment compared to international markets. However, weaknesses included unaffordable costs for most South Africans, limited training opportunities for reproductive medicine subspecialists, and gaps in regulatory enforcement. Opportunities were identified in expanding fertility tourism, leveraging the recent legalisation of preimplantation sex selection, and adopting operational innovations such as low-cost in vitro fertilisation (IVF). Major threats included political inertia, rising operational costs, and professional emigration.

**Discussion:**

These findings highlight the sector's strong global competitiveness alongside significant domestic structural challenges. Based on stakeholder insights, this study proposes strategic interventions to expand specialist training, modernise donor practices, establish statutory regulation, and position South Africa more effectively as a global leader in fertility healthcare.

## 1 Introduction

A complex and evolving landscape shapes the fertility healthcare sector in South Africa. While assisted reproductive technologies (ARTs) have become more accessible and in demand, research on the specific dynamics and challenges within South Africa's fertility healthcare sector remains limited. A detailed SWOT (Strengths, Weaknesses, Opportunities, Threats) analysis can provide valuable insights into the industry's current state and future potential.

Among the available studies, the African Network and Registry for ART (ANARA) provides an overview of the South African sector within the broader African region. According to ANARA's report on 2018 and 2019 (1) South Africa performed 8,003 ART procedures in 2018 and 8,663 in 2019, across 15 and 19 centres respectively, positioning the country as a leader in ART in sub-Saharan Africa. However, the report also highlights challenges, such as high rates of multiple births resulting from the common practice of transferring multiple embryos. This increases the risk of preterm deliveries and other perinatal complications. The findings suggest the need to optimise ART practices, such as encouraging single embryo transfers (SET), to enhance safety while maintaining high success rates.

Infertility affects an estimated 8–12.5% of couples globally and up to 23% in sub-Saharan Africa, with South Africa facing a prevalence of 15%–20% and an annual need for 76,500 ART cycles, of which only 6.4% is met due to limited access (2). High costs exacerbate this gap, with public-sector IVF cycles costing R40,000–R50,000 and private-sector cycles ranging from R70,000 to R100,000, often requiring out-of-pocket payments due to limited medical aid coverage (3). In 2012, public sector costs were significantly lower, ranging from R7,000 for low-cost IVF to R28,000 for private patients, while private sector IVF averaged R36,368, with laboratory fees accounting for up to 38% of expenses (4). These costs, combined with long waiting times of 8–12 months in public facilities, restrict access, particularly for lower-income groups (2, 3). The sector also grapples with a shortage of reproductive medicine subspecialists, driven by limited training capacity and physician migration to developed countries in search of better opportunities (5).

Yet, the South African fertility healthcare sector is a magnet for fertility tourism. Patients from Namibia, Zimbabwe, Cameroon, the UK, Germany, Norway, and Australia seek ART due to unavailable services, mistrust in local clinics, or restrictive regulations in their home countries (6).

Additionally, a survey of egg donors in South Africa provides critical insights into the ethical and regulatory issues faced by the sector (7). The study found that while 95% of donors reported a positive experience, 7% did not recall receiving sufficient information about potential medical risks associated with the donation (7). Furthermore, many donors expressed dissatisfaction with the amount of compensation provided (7). These findings point to the need for improvements in both the ethical management of donor relationships and the regulatory framework governing ART practices.

This article aims to conduct a comprehensive SWOT analysis of the South African fertility healthcare sector. It will assess the sector's strengths, such as its advanced medical infrastructure, professional expertise, and a progressive regulatory framework that attracts diverse international patients, as well as its weaknesses, including regulatory inconsistencies, concerns about donor management, high treatment costs, limited public-sector access, and shortages of trained specialists. Furthermore, this analysis will explore growth opportunities, particularly in areas such as fertility tourism and low-cost ART innovations, while also addressing external threats, including ethical controversies and potential market saturation, as well as economic barriers. The goal is to provide a comprehensive understanding of the sector and its future trajectory.

## 2 Methodology

### 2.1 Data collection

This article is based on qualitative research conducted through a series of semi-structured interviews with key stakeholders operating within the private fertility healthcare sector in South Africa. Participants included representatives of a national fertility society, as well as directors of private fertility clinics, genetics laboratories, sperm and egg banks, and egg donation agencies, drawn from diverse geographic locations across South Africa. The purpose of these interviews was to explore the roles, interactions, and challenges faced by different actors within the industry and to inform a SWOT analysis based on the insights gathered, with particular attention to market dynamics. Twelve interviews were conducted with individuals occupying senior leadership positions within the sector. Focusing on private-sector actors was a deliberate methodological choice, as public-sector clinics operate under substantially different funding, governance, and incentive structures and were therefore not considered analytically relevant to the study's market-oriented objectives. While the fertility healthcare landscape includes other stakeholders such as embryologists, psychologists, fertility lawyers, and medical suppliers, these roles were not systematically represented in the sample, a limitation acknowledged. The authors considered interviewing fertility patients and gamete donors; however, this approach was not pursued. Patients and donors typically engage with the fertility healthcare sector on an episodic basis. They are unlikely to possess detailed knowledge of its internal organisational structures, regulatory environment, or market operations.

Each interview lasted approximately 90 min and was conducted with participants' informed consent, including explicit permission for audio recording. Participants were informed about the study's purpose, the use of recordings, and their right to withdraw at any time. Interviews were audio-recorded and transcribed verbatim to ensure accuracy. All data are securely stored and will be retained for 5 years in accordance with the University of KwaZulu-Natal's research ethics guidelines, after which they will be permanently destroyed. Given the small and elite nature of the sample, particular care was taken to minimise the risk of deductive disclosure through anonymisation, including the removal of direct identifiers, the use of pseudonyms, and the generalisation or omission of contextual details that could reasonably enable participant identification.

The interview schedule was designed to elicit insights into stakeholder roles, regulatory and market environments, ethical and operational challenges, and patterns of interaction among sector actors, particularly in relation to donor and patient management and broader market dynamics. Data saturation was reached, with recurring themes emerging across interviews, indicating that the dataset was sufficient to address the study's research objectives.

### 2.2 Data analysis

A thematic approach was employed to analyse the transcribed interview data. Each transcript was reviewed in detail, and key themes relevant to a SWOT analysis of the South African fertility healthcare sector were identified. The findings reflect a diversity of stakeholder perspectives, with some themes, such as donor anonymity, emerging strongly from specific interviews rather than representing universal consensus across all participants.

To assist with the integration and preliminary organisation of the data, the researchers utilised ChatGPT-4, a large language model developed by OpenAI. Anonymised transcripts were uploaded one by one, and prompts were designed to elicit structured thematic summaries aligned with the SWOT framework. Example prompts included requests to identify recurring strengths, weaknesses, opportunities, and threats, as well as patterns relating to donor recruitment, regulatory challenges, and market dynamics. The model was instructed to summarise patterns evident in the data without introducing new interpretations.

Recognising the limitations inherent in AI-generated outputs—such as the potential to overlook contextual nuance, overgeneralise diverse viewpoints, or introduce interpretive bias—the researchers undertook a rigorous manual verification process. Each AI-generated thematic summary was independently reviewed and compared against the original transcripts to confirm accuracy, resolve discrepancies, and eliminate unsupported inferences.

By combining AI-assisted efficiency in initial pattern identification with human-led validation, the researchers aimed to preserve analytical depth while improving the management of large, diverse interview materials. The resulting thematic analysis informed the SWOT framework presented in the Results section and provided the foundation for the more detailed exploration of selected issues in the Discussion section.

### 2.3 Ethical considerations

The study adhered to the ethical guidelines established by the University of KwaZulu-Natal's Biomedical Research Ethics Committee (approval number BREC/00005424/2023). Informed consent was obtained from all participants prior to the interviews, and all data were anonymised to ensure participants’ confidentiality. All local copies of the data will be deleted upon completion of the study.

### 2.4 Limitations

This study acknowledges certain limitations. First, the relatively small number of interviews may not fully capture the diversity of perspectives across the fertility healthcare sector, particularly from roles such as embryologists, psychologists, and fertility lawyers. Additionally, the data are based on interviewees' subjective insights, which may introduce bias. Despite these limitations, the richness of the qualitative data provides valuable insights into the dynamics and challenges of the South African fertility healthcare sector, particularly given the limited research on this topic.

## 3 Results

The analysis of interviews with key stakeholders in the South African fertility healthcare sector reveals several insights into the industry's current dynamics. These insights are grouped according to the SWOT framework, based on the interviewees' perspectives and experiences.

### 3.1 Strengths

The interviews highlighted the firm foundation of the South African fertility healthcare sector, primarily driven by advanced medical infrastructure and highly skilled professionals. Stakeholders emphasised that the sector benefits from world-class reproductive technologies, placing it on par with international standards. Additionally, the expertise of reproductive medicine subspecialists, embryologists, and supporting professionals was repeatedly noted as a key strength. These professionals, some of whom have received international training, make a significant contribution to the sector's ability to deliver high-quality care to both local and international patients. Stakeholders also praised the sector's efficient communication and quick turnaround times compared to international counterparts, enhancing its competitive edge. As one stakeholder noted, “[…] the South African clinics are very, very good at communicating. They are very quick, they are very thorough, they are, for the most part, very ethical.” As one stakeholder emphasised, the sector plays a vital role in “helping people realise their dreams and continue their heritage,” underscoring its societal significance.

Another notable strength identified is South Africa's growing reputation as a destination for fertility care. The relatively lower costs of fertility treatments compared to many developed countries, combined with high success rates, make the country an attractive option for patients seeking affordable but high-quality services. The sector's diverse donor pool and progressive regulatory framework further enhance its appeal, attracting a wide range of international patients, including same-sex couples and single women. One stakeholder highlighted, “We have an amazing selection of young women who are egg donors in this country because we are so multicultural, multi-diverse, so that there is someone for everyone.”

Another emphasised the regulatory advantage, stating, “about almost half of my business were intended parents that came from abroad, especially Australia. Australia was our biggest international market.” The sector's ability to cater to a global market, particularly through fertility tourism, was recognised as a significant asset. Additionally, the variety of clinic sizes, from extensive facilities to smaller practices, enhances patient choice. As one stakeholder explained, “we have access to a large number of clinics to be able to go to have treatment done […] so I think that's a benefit.” The Southern African Society of Reproductive Medicine and Gynaecological Endoscopy (SASREG) was also viewed by half the participants as a strength, given its role in providing guidance and certification to clinics in the absence of robust government regulation in these areas.

### 3.2 Weaknesses

The most serious weakness appears to be the limited number of reproductive medicine subspecialists available to meet the growing demand for services. Training opportunities for gynaecologists to subspecialise in fertility medicine are severely restricted, with such programmes currently offered at only two locations in the country, each with limited capacity. Consequently, the supply side of fertility healthcare services remains constrained, even as demand increases both due to the growth of the South African population and the sector's expansion to serve international patients. This shortage is closely linked to—and likely a major contributor to—what we regard as the greatest weakness of the South African fertility healthcare sector: the unaffordability of treatments for most South Africans. As one stakeholder observed, “the cost is prohibitive to a lot of the population, which is always sad in medical treatments.”

Another key weakness identified relates to the recruitment of gamete donors. Many stakeholders expressed concern about the challenges of recruiting donors. High marketing and administrative costs for donor agencies complicate recruitment efforts, with one stakeholder stating, “We spend an absolute fortune marketing and advertising.” Patient preferences for imported sperm to ensure social and genetic distance from the donor, and challenges in recruiting sperm donors from some minority groups, particularly from Indian and Muslim communities, further hinder local donor programmes. In the case of egg donation, the time commitment and invasive nature of the donation process are significant deterrents. Furthermore, concerns exist regarding the legal framework governing donor compensation and anonymity, which adds complexity to the recruitment process.

### 3.3 Opportunities

The interviews highlighted several growth opportunities for the fertility healthcare sector in South Africa. One of the most prominent opportunities identified was the expansion of fertility tourism. Many stakeholders indicated that South Africa's competitive advantage—offering high-quality care at lower costs than many developed countries—presents a significant opportunity to attract more international patients. Cape Town has emerged as a key destination, drawing patients from Europe, Australia, and Africa. Clinics are enhancing accessibility through logistical support, such as interpreters and dedicated sections of their websites for international patients. As one stakeholder emphasised, “South Africa has got a good name and […] our costs are so much less than overseas,” highlighting the sector's global appeal.

Another key opportunity lies in adopting new reproductive technologies and operational innovations. Stakeholders expressed optimism about the potential for innovations, such as genetic screening and embryo selection, to enhance success rates and provide more personalised treatments. However, some noted the high cost of these technologies. The implementation of practices like SET was also seen as a way to enhance safety by reducing the risks associated with multiple births, which remains a concern in the sector. Additionally, operational advancements, such as online psychological appointments and inter-clinic collaboration for donor scans, are streamlining services.

While ethical debates on non-medical pre-implantation sex selection persist, the Pretoria High Court in *Surrogacy Advisory Group v Minister of Health* found insufficient evidence of harm to justify prohibiting it, thereby legalising it under South Africa's Constitution (8–10). This landmark ruling, dating from 2022, affirmed that non-medical pre-implantation sex selection falls within the constitutionally protected realm of reproductive autonomy, encompassing not only the decision to have children but also the personal choices about family composition and the conditions of parenthood [sections 12(2) and 27]. By prioritising individual rights to bodily and psychological integrity and privacy, the decision empowers prospective parents with greater control over their reproductive journeys. This development—which now allows clinics to offer preimplantation sex selection without inquiring into patients' motivations, whether medical or otherwise—was highlighted by some of the stakeholders as an attractive new service for patients, enhancing opportunities for personalised and inclusive family planning.

### 3.4 Threats

According to stakeholders, a primary threat to the sector is political, specifically the South African government's perceived lack of prioritisation of fertility healthcare—particularly in policy-making and implementation. For example, although the South African Department of Health was mandated by law over a decade ago to establish a central database for donor information, this mandate has yet to be implemented. This is problematic for the sector—as one stakeholder explained, “there's no central place […] that you can actually as agencies go and look at.” While SASREG has stepped into the regulatory vacuum left by the government, it remains a voluntary organisation without statutory power to enforce its guidelines. Yet it plays a vital role in many respects, including setting standards and accrediting clinics. Stakeholders noted that some clinics operate without accreditation, often run by practitioners who lack specialised qualifications, thereby undermining the quality of care. Furthermore, SASREG's structure was criticised for underrepresenting embryologists, nurses, and other stakeholders, limiting its inclusivity. In the absence of broader inclusivity, SASREG lacks the legitimacy to serve as a comprehensive self-regulatory body for the sector.

The lack of government support could also hinder the operationalisation of low-cost IVF technologies in the public healthcare system, which would require a strong political will and the retention of expert fertility healthcare professionals. Although SASREG has made inroads with private medical insurers to cover certain aspects of fertility treatment at accredited clinics, these aspects remain inaccessible to most South Africans.

The failure of active regulation also exacerbates potential legal and ethical risks, such as South African women being paid handsome sums to travel abroad for egg donation. Given that South African legislation only permits compensation for reasonable expenses, such arrangements could expose the sector to legal scrutiny. Moreover, stakeholders expressed concern that public attention to complications—such as a woman returning with ovarian hyperstimulation syndrome—could trigger a legislative backlash, further constraining the sector.

High costs of imported medical supplies and laboratory consumables, influenced by exchange rate fluctuations, also pose a threat to affordability. As one industry representative explained, “99% of the stuff that we buy in our industry, especially the lab, is imported […] what makes access to fertility treatment more difficult in general, or should I say, it is more expensive.”

Additionally, the emigration of skilled fertility healthcare professionals, often motivated by political and economic concerns, poses a serious threat to the sector's future viability. This situation is further complicated by the limited training opportunities available to new reproductive medicine subspecialists.

Lastly, stakeholders highlighted the absence of insurance for embryologists, noting the vulnerability this creates in the event of litigation. As one stakeholder warned, “There is going to be a huge lawsuit […] There is going to be a fight about whether medico-legally, who is the one that is ultimately responsible for patient care, when the clinician doesn't know what is done in the lab.”

### 3.5 Conclusion of the results

In conclusion, the interviews reveal that the South African fertility healthcare sector is characterised by remarkable strengths—particularly in its skilled workforce, advanced technologies, and growing international reputation—but is equally constrained by serious challenges related to affordability and workforce shortages. Opportunities for expansion, innovation, and broader access are clear, yet the sector faces significant threats from political inertia, economic vulnerabilities, and professional emigration. As one industry representative noted, “[w]e are adaptive to what is happening […] worldwide—I think that is fantastic,” underscoring the sector's resilience. Addressing these challenges while strategically leveraging opportunities will be essential to ensuring the sector's sustainable growth and success, supported by a diverse ecosystem of stakeholders, including clinicians, embryologists, psychologists, and fertility lawyers.

## 4 Discussion

Insights from diverse stakeholders offer a nuanced, forward-looking understanding of the South African fertility healthcare sector. They reveal strengths such as global competitiveness and growth potential in fertility tourism, alongside critical structural challenges, particularly in access, talent development, and regulatory coherence. Building on these findings, this discussion proposes strategies to address four key issues vital to the sector's sustainable advancement.

### 4.1 Expanding training and talent development

A consistent theme across stakeholder interviews was the urgent shortage of reproductive medicine subspecialists in South Africa. The limited number of training facilities—currently only two—and their restricted intake capacities severely constrain the sector's growth. This shortage not only limits service availability but also contributes significantly to the high costs of fertility treatments, thus restricting access for most South Africans.

To address this, training opportunities must be dramatically expanded. A trial model for reproductive medicine subspecialty training, implemented across 12 accredited decentralised units, demonstrates the feasibility of a two-year full-time or four-year part-time fellowship, supported by private-sector funding and certified by the Health Professions Council of South Africa (5). This model, involving private practice obstetricians and gynaecologists, offers a blueprint for scaling training through new centres (5). Public-private partnerships could be fostered to establish additional accredited training centres across the country, leveraging funding from hospital groups, medical insurers, and per-cycle ART donations to ensure sustainability (5). Furthermore, part of the community service requirements for qualified reproductive medicine subspecialists could include mentoring trainees, thereby integrating training into existing practice environments. As stakeholders noted, the sector's sustainability depends on cultivating a new generation of specialists who are not only technically competent but also more representative of South Africa's diverse demographics. Investing in structured mentorship and expanding the training infrastructure is crucial to ensuring both affordability and accessibility in the long term.

### 4.2 Strengthening regulatory oversight and accountability

Stakeholders unanimously identified the absence of comprehensive statutory regulation as a significant weakness. Currently, the sector relies on voluntary SASREG accreditation. Although, SASREG provides essential clinical guidance, its lack of statutory authority results in inconsistent standards and limited enforcement capabilities.

We recommend establishing a dedicated statutory regulatory authority for fertility healthcare, similar to the United Kingdom's Human Fertilisation and Embryology Authority (HFEA). Such a body should have the power to set uniform clinical, ethical, and operational standards; conduct regular audits; and maintain an up-to-date central donor database—a legal requirement that remains outstanding. This authority could address current gaps, such as the lack of national guidelines for pathogen screening, particularly for HIV, which complicates safe ART delivery (4). Fiscal evidence suggests that integrating ART into national health insurance could generate a 5.64 return on investment through lifetime tax contributions, supporting policy reforms to recognise infertility as a public health issue (2). Streamlining referral processes and reducing public sector waiting times would further enhance access to healthcare (3). Importantly, its governance structure must ensure broad sectoral representation, including clinics, agencies, donors, patients, and supporting professionals. Stakeholders generally welcomed this idea, noting that it would foster greater transparency, accountability, and public trust, ultimately safeguarding both local and international patients.

### 4.3 Modernising donor practices: From anonymity to informed choice

Despite legal clarification that South African law permits open-identity gamete donation with the donor's written consent (8), the sector continues to operate predominantly under outdated assumptions favouring donor anonymity. This restricts donors' and intended parents' options, including those who may prefer identity-release arrangements, and limits the sector's alignment with international best practices.

Clinics, banks, and agencies should revise their systems to allow for informed choice, enabling donors and recipients to select either an anonymous or an identity-release donation model. Practical system changes must accompany this shift, including the ability to manage various consent models and to ensure clear communication with all parties involved. Cultural and religious barriers, such as prohibitions on donor gametes among some Muslim couples, highlight the need for culturally sensitive counselling to support informed decision-making (3). Interviews revealed that recipients increasingly desire greater transparency—for example, access to adult photos of donors—while respecting the boundaries of anonymity in cases where the donor chose to remain anonymous. Updating donor practices will align the sector with evolving ethical norms and enhance its global competitiveness.

### 4.4 Strategically leveraging fertility tourism

Fertility tourism represents one of the most promising growth areas for the South African fertility healthcare sector. High-quality treatments offered at comparatively lower costs, progressive regulations accommodating diverse family structures, and a multicultural donor pool give South Africa a distinct competitive advantage. Cape Town, in particular, has established itself as a premier destination, attracting patients from Europe, Australia, and across the African continent.

Although not always fully appreciated by the stakeholders during the interviews, we suggest that a significant recent development is the legalisation of non-medical pre-implantation sex selection (9–11). This service, unavailable in many other jurisdictions due to ethical concerns, but supported by South Africa's constitutional commitment to reproductive autonomy, allows intended parents to select the sex of their child before transfer *in utero*. As such, it is likely to become a major attraction for international patients seeking greater reproductive choice. We suggest that this legal development remains underappreciated among stakeholders and can also be leveraged to drive inbound fertility tourism further.

To maximise this opportunity, comprehensive travel and treatment packages should be developed, integrating medical services with accommodation, transport, interpreter services, and dedicated online platforms tailored for international patients. Partnerships with the tourism industry could amplify these offerings, creating seamless experiences for fertility tourists.

## 5 Conclusion

The South African fertility healthcare sector is built on a strong foundation of clinical expertise, advanced infrastructure, and international appeal. Yet its future success will depend, above all, on expanding access—making fertility care more affordable, accessible, and ethically grounded for all who seek it. Addressing challenges such as talent shortages, regulatory gaps, and outdated donor practices is essential to achieving this goal. Recent legal developments, including the legalisation of preimplantation sex selection, create significant opportunities to enhance the sector's global competitiveness. By prioritising reforms that advance access to innovation, South Africa can realise its potential as a global leader in inclusive, patient-centred fertility care.

## Data Availability

The raw data supporting the conclusions of this article will be made available by the authors, without undue reservation.
